# Assessing serial recall as a measure of artificial grammar learning

**DOI:** 10.3389/fpsyg.2024.1497201

**Published:** 2024-12-18

**Authors:** Holly E. Jenkins, Ysanne de Graaf, Faye Smith, Nick Riches, Benjamin Wilson

**Affiliations:** ^1^Department of Education, University of Oxford, Oxford, United Kingdom; ^2^Faculty of Social and Behavioural Sciences, University of Amsterdam, Amsterdam, Netherlands; ^3^School of Education, Communication and Language Sciences, Newcastle University, Newcastle upon Tyne, United Kingdom; ^4^Department of Psychology, Emory University, Atlanta, GA, United States; ^5^Emory National Primate Research Center, Emory University, Atlanta, GA, United States

**Keywords:** implicit learning, statistical learning, sequence learning, artificial grammar learning, chunking, recall

## Abstract

**Introduction:**

Implicit statistical learning is, by definition, learning that occurs without conscious awareness. However, measures that putatively assess implicit statistical learning often require explicit reflection, for example, deciding if a sequence is ‘grammatical’ or ‘ungrammatical’. By contrast, ‘processing-based’ tasks can measure learning without requiring conscious reflection, by measuring processes that are facilitated by implicit statistical learning. For example, when multiple stimuli consistently co-occur, it is efficient to ‘chunk’ them into a single cognitive unit, thus reducing working memory demands. Previous research has shown that when sequences of phonemes can be chunked into ‘words’, participants are better able to recall these sequences than random ones. Here, in two experiments, we investigated whether serial visual recall could be used to effectively measure the learning of a more complex artificial grammar that is designed to emulate the between-word relationships found in language.

**Methods:**

We adapted the design of a previous Artificial Grammar Learning (AGL) study to use a visual serial recall task, as well as more traditional reflection-based grammaticality judgement and sequence completion tasks. After exposure to “grammatical” sequences of visual symbols generated by the artificial grammar, the participants were presented with novel testing sequences. After a brief pause, participants were asked to recall the sequence by clicking on the visual symbols on the screen in order.

**Results:**

In both experiments, we found no evidence of artificial grammar learning in the Visual Serial Recall task. However, we did replicate previously reported learning effects in the reflection-based measures.

**Discussion:**

In light of the success of serial recall tasks in previous experiments, we discuss several methodological factors that influence the extent to which implicit statistical learning can be measured using these tasks.

## Introduction

Implicit statistical learning refers to the ability to detect and extract information from the environment. Critically, this occurs without conscious awareness or any intention to learn (Conway, 2020). Although implicit statistical learning is relevant for many aspects of cognition, it plays a particularly important role in supporting language acquisition and processing, given that knowledge of many aspects of language is acquired implicitly and without conscious effort (Kidd, 2012). For example, infants have been shown to use statistical regularities to detect linguistic features such as word boundaries within a continuous speech stream (Saffran et al., 1996; Aslin et al., 1998; Saffran, 2002). Furthermore, artificial grammar learning paradigms have also shown that implicit statistical learning is important for acquiring knowledge relating to grammatical structure, including the relationships between words and phrases (Reber, 1967; Saffran, 2002; Saffran et al., 2008; Gómez and Gerken, 2000). Over the last 50 years, a wide range of studies have approached these questions in the related fields of implicit learning and statistical learning (for a review of this literature, see Perruchet and Pacton, 2006; Christiansen, 2019). Moreover, these processes have been addressed using a wide range of different tools. These include behavioural approaches (such as head turn preference paradigms in infants (e.g., Saffran et al., 1996), grammaticality judgement tasks (e.g., Reber, 1967, and see below) and eye-tracking (e.g., Wilson et al., 2015), neuroimaging and brain stimulation techniques (e.g., fMRI, Friederici et al., 2006; EEG, Hagoort and van Berkum, 2007; TMS, Ambrus et al., 2020; TDCS, Smalle et al., 2017), and modelling (McCauley and Christiansen, 2014). These paradigms have been important in determining the extent to which implicit statistical learning supports learning of grammatical structure.

Traditionally, artificial grammar learning paradigms have been used to investigate the role of implicit statistical learning in language acquisition. The experiments typically begin with an exposure phase, where participants are presented with sequences that follow the rules of an artificial grammar. In this exposure phase, participants are asked to attend to the sequences without making any responses, but they are not informed of any rules underlying the sequences. Following exposure, participants complete a testing phase, usually in the form of a grammaticality judgement task. In this task, participants are informed that the sequences in the exposure phase followed a pattern and asked to classify subsequent sequences as “following” or “breaking” this pattern. If implicit statistical learning has taken place, then participants should be better able to classify the sequences in the testing phase despite not having conscious awareness of the pattern. A considerable number of studies have demonstrated that implicit statistical learning is involved in the learning of grammatical relationships using these tasks (Batterink et al., 2015; Curran, 1997; Dienes and Altmann, 1997; Reber and Squire, 1994; Scott and Dienes, 2008; Tunney and Altmann, 2001; Willingham and Goedert-Eschmann, 1999).

However, although the aim of artificial grammar learning paradigms is often to measure learning that is not consciously accessible, grammaticality judgement tasks may not be the most appropriate method of testing implicitly acquired information or implicit knowledge (Christiansen, 2019). Grammaticality judgement tasks are an example of a ‘reflection-based’ task, which rely on the participants using conscious reflection to make explicit decisions about what has been learned. Therefore, these tasks can only measure knowledge that learners can access explicitly and may not accurately reflect *implicit* knowledge. Furthermore, because reflection-based tasks typically only assess learning after the exposure phase—after implicit statistical learning has taken place—they are somewhat limited in the information that they can provide about *how* learning takes place (Siegelman, 2020).

More recently, there has been some focus on developing more implicit, ‘processing-based’ tasks which do not require conscious awareness, and are therefore less likely to reflect explicit processes (e.g., Isbilen et al., 2017; Misyak et al., 2010). Processing-based tasks also offer additional benefits over traditional reflection-based tasks. For example, they allow learning to be measured ‘online’ over the course of the experiment, as participants are required to make responses while they are learning as opposed to in a separate testing phase following exposure. To avoid conscious processing, these tasks typically assess learning indirectly, by measuring other variables that are facilitated by implicit statistical learning. For example, previous research has used serial reaction time tasks to measure learning, by demonstrating that participants respond more quickly to predictable sequences than to unpredictable sequences (Nissen and Bullemer, 1987; Misyak et al., 2010, although see Jenkins et al., 2024).

Implicit statistical learning also facilitates recall of predictable sequences through a cognitive process called ‘chunking’; frequently co-occurring items can be combined into a single cognitive unit to reduce demands on working memory (Miller, 1956). This process of chunking occurs both during language acquisition (when infants are learning to detect word boundaries in a speech stream; Saffran et al., 1996; Aslin et al., 1998) and processing (where speech must be chunked and passed on to higher levels of linguistic representation, e.g., from phonemes to words, then to phrases and sentences; Christiansen and Chater, 2016). Based on this, participants should be able to chunk, and later recall, predictable sequences more easily than unpredictable sequences, as only predictable sequences contain frequently co-occurring elements. Note that, critically, while these tasks do require attention to perceive and hold the stimuli in working memory, this attention is directed to the stimuli sequences themselves, and not to the underlying grammatical rules. This is distinct from grammaticality judgement tasks, which not only require attention to the stimuli, but also direct participants to search for patterns and rules. Therefore, processing-based measures such as serial recall tasks represent a valuable tool to assess statistical learning more implicitly. This effect has been demonstrated across a range of tasks, including visuo-motor learning tasks (Conway et al., 2007), auditory (Isbilen et al., 2017; Kidd et al., 2020) and visual statistical learning tasks (Isbilen et al., 2020), and even in natural language (McCauley and Christiansen, 2015).

Although previous research has shown that serial visual recall can be used as a processing-based measure of implicit statistical learning, more information is needed to determine what constraints exist on measuring learning using these tasks. Specifically, most prior tasks have used the same trisyllabic stimuli as the seminal statistical learning paradigm introduced by Saffran et al. (1996), in which the ‘words’ are comprised of syllables that always co-occur (Isbilen et al., 2017, 2020, 2022; McCauley and Christiansen, 2015). However, the role of implicit statistical learning in language has been shown to extend beyond learning of word boundaries; similar processes have been shown to underlie learning of more variable relationships *between* words in artificial grammars (Gomez and Gerken, 1999; Newport and Aslin, 2004). Crucially, this variability precludes forming stimuli into invariant ‘chunks’ (as is possible with the triplet ‘words’ used previously). This raises the question of whether processing-based tasks using serial recall can measure learning of these more variable relationships, and thus how broadly applicable might these approaches be.

**Figure 1:**
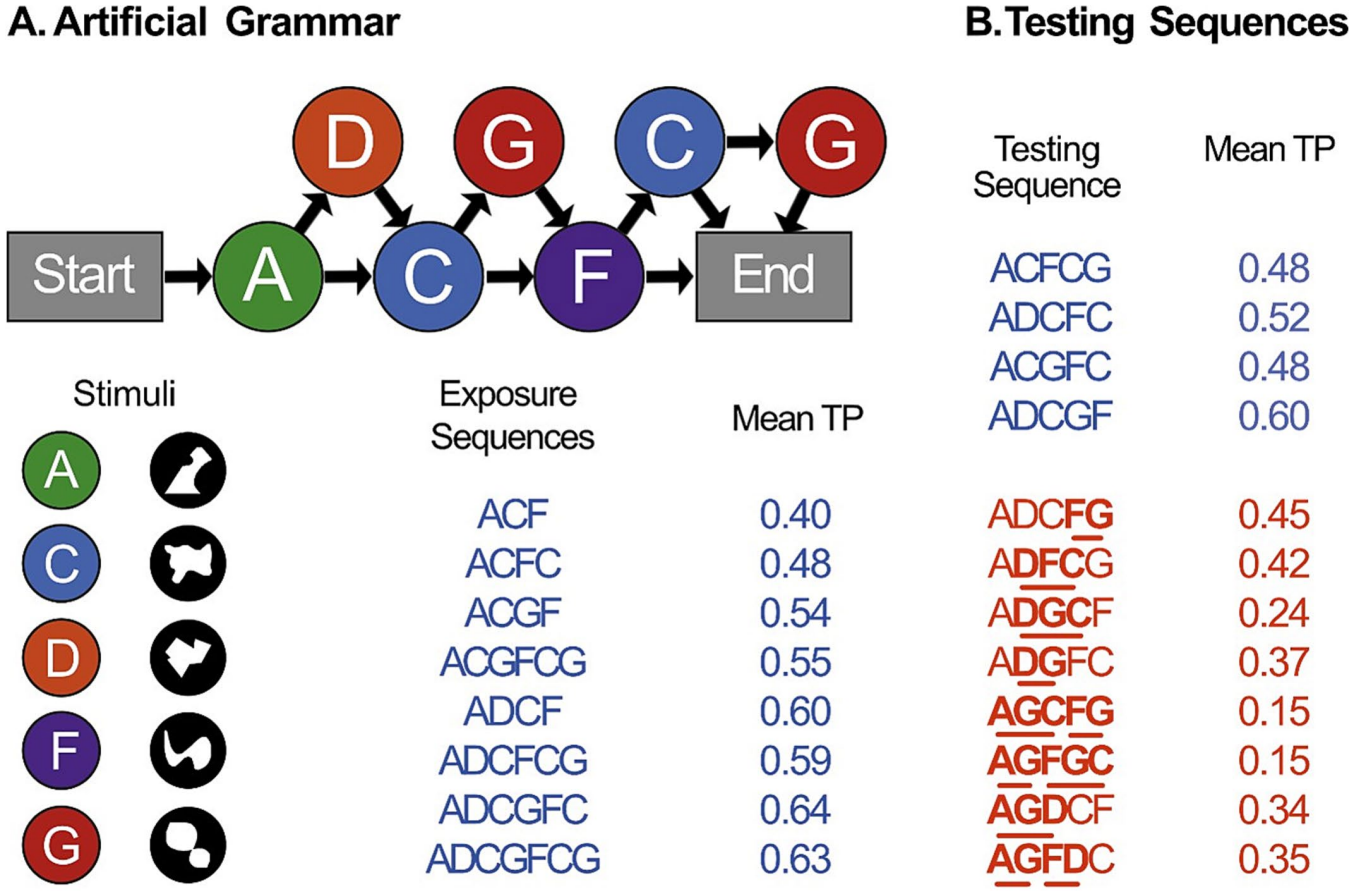
Artificial grammar and stimuli. **(A)** Illustration of the artificial grammar, stimulus elements, and the exposure sequences used in Experiments 1 and 2. Sequences are produced by following the arrows from the start to the end. The grammar contains 5 elements which are represented by abstract shapes. For exposure, we used all possible grammatical sequences except for those which were 5 elements long, which were kept for testing. **(B)** The testing sequences consisted of 4 grammatical sequences (shown in blue), each of which was repeated twice per block, and 8 ungrammatical sequences (shown in red), each presented once per block. Underlined bigrams represent ungrammatical transitions, not allowed by the artificial grammar. The average transitional probability (TP) of each sequence is reported.

In two experiments (a lab-based experiment, and a subsequent online replication), we aimed to investigate whether serial visual recall could be used to effectively measure learning of the between-word relationships found in artificial grammar. These grammars have previously been used in both the auditory and visual modalities in humans and nonhuman primates (Milne et al., 2018; Saffran et al., 2008; Saffran, 2002; Wilson et al., 2013; Wilson et al., 2015). To do this, we adapted the design and stimuli of a previous AGL study (Milne et al., 2018), and integrated it with a novel Visual Recall task. The artificial grammar consists of five stimuli, in this case abstract visual shapes (see Figure 1). These stimuli were presented serially in sequences generated by an artificial grammar. We first exposed participants to sequences of “grammatical” sequences. Following this, the participants were presented with novel testing sequences, and, after a brief pause, were asked to recall the sequence by clicking on the visual symbols on the screen in order. Participants completed four blocks of recall testing in total. In both experiments we predicted that participants would show learning in the Visual Recall task, evidenced by a greater increase in recall accuracy of grammatical sequences compared to ungrammatical sequences across blocks.

In both experiments, following the Visual Recall task, participants completed two reflection-based tasks: a traditional Grammaticality Judgement task and a Sequence Completion task. In the Grammaticality Judgement task, participants were once again exposed to grammatical sequences. They were then told that the sequences followed certain patterns. They were presented with testing sequences, and asked whether each sequence followed the same patterns as the exposure sequences, or whether they did not. In the Sequence Completion, participants once again completed a short exposure phase. They were then presented with incomplete sequences and asked to complete them by selecting the missing symbol from an array of options on the screen. The Sequence Completion task was included to further assess the extent to which any sequence knowledge that was obtained was available to consciousness, as the ability to complete a partial sequences would suggest more explicit knowledge of the structure (Destrebecqz and Cleeremans, 2001; Wilkinson and Shanks, 2004). Across all experiments, we predicted that we would see evidence of learning across the both the processing-based tasks and the reflection-based tasks. Correlations between the processing- and reflection-based tasks would suggest similar (likely explicit) processes in both cases. By contrast, a lack of correlation would suggest that these tasks measure different learning systems.

## Methods

### Participants

In both experiments, participants were pre-screened to include native English speakers and exclude participants who had language disorders, as previous research has suggested that there may be deficits in implicit statistical learning in these populations (Folia et al., 2008; Hsu and Bishop, 2014; Obeid et al., 2016). All participants had normal or corrected-to-normal vision and hearing, and participants were not excluded based on their ability to speak any additional languages.

In Experiment 1, 22 adult participants (15 female, 7 male, mean age = 30.1) were recruited from the Institute of Neuroscience participant pool at Newcastle University. Ethics was approved by the Faculty of Medical Sciences Ethics Committee at Newcastle University.

Experiment 1 was carried out in-person prior to the COVID-19 pandemic. Due to the pandemic lockdown in the UK, further in-person data collection was not possible. Therefore, we re-ran the experiment online. In Experiment 2, 43 participants (26 female, 17 male; mean age = 30.98 years) were recruited using Prolific, an online recruitment platform. An additional 7 participants completed the experiment but were excluded from analysis for failing attention checks. Ethics was approved by Emory University IRB.

### Stimuli

**Figure 2:**
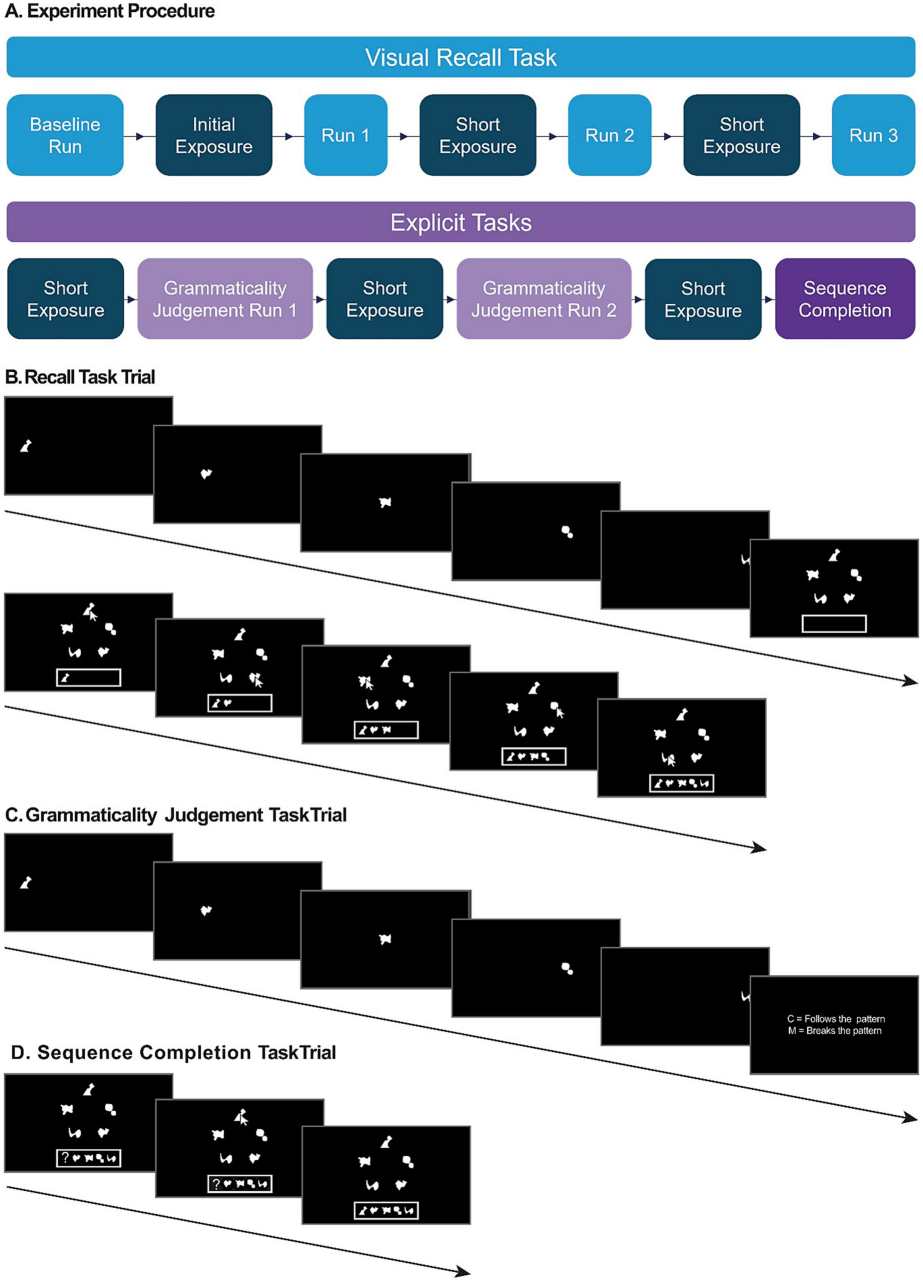
Procedure and trial design. **(A)** Experiment procedure. Participants completed three tasks in this experiment, first the Visual Recall task followed by the more explicit Grammaticality Judgement and Sequence Completion tasks (see Methods). **(B)** Visual Recall task. In each trial, a sequence of 5 shapes was presented serially across the screen. Each shape was presented for 450 ms, and each shape was separated by a 300 ms inter-stimulus interval. After the sequence had been displayed, there was a 1,000 ms retention period. Following this, participants were presented with all 5 stimuli simultaneously on the screen in randomized positions. Participants were asked to recreate the sequence by clicking on the elements in order. Each trial was separated by a 1,500 ms inter-trial interval. **(C)** Grammaticality Judgment task. Participants were presented with grammatical and ungrammatical sequences. Following each sequence, participants pressed one of two keys on the keyboard to indicate whether they thought that the sequence followed the same pattern as the sequence they had seen previously or not. Each trial was separated by a 1,500 ms inter-trial interval. **(D)** Sequence Completion task. In each of the 24 trials, participants were presented with a 5-element long sequence in which one of the elements was replaced by a question mark. Participants were asked to complete the sequence by clicking on the desired shapes to fill in the gap. Each trial was separated by a 1,500 ms inter-trial interval.

The stimuli sequences were generated using an artificial grammar developed by Saffran (2002) and Saffran et al. (2008), using abstract shapes inspired by previous artificial grammar stimuli (Conway and Christiansen, 2006; Milne et al., 2018; Osugi and Takeda, 2013; Seitz et al., 2007). The grammar consisted of 5 elements (A, C, D, F, G), each represented by an abstract white shape (200 × 200 pixels) on a black background (see Figure 1). The Visual Recall task was split into exposure and testing phases. The exposure phases consisted of 8 different grammatical sequences presented 8 times, totalling 64 sequences (see Figure 1). This included all possible grammatical sequences, except those which were 5 elements long, which were not presented during the exposure phase so they would remain novel to the participants in the testing phase. In the testing phase, participants were presented with 5-element-long grammatical and ungrammatical sequences, none of which had previously appeared in the exposure phase. There were 4 grammatical sequences and 8 ungrammatical sequences (see Figure 1), so each grammatical sequence was presented twice per phase to ensure the number of grammatical and ungrammatical sequences was balanced. The ungrammatical sequences contained fewer frequently co-occurring transitions (and as such had lower average transitional probabilities (TP) and associative chunk strengths (ACS)) and are therefore harder to chunk. (For discussion of the relationship between TPs and ACS in the field of implicit statistical learning, see Perruchet and Pacton, 2006). The same sequences from the testing phase were used in the Grammaticality Judgement task. In the Sequence Completion task, participants were presented with grammatical sequences with one stimulus element replaced by a question mark. They were required to select the stimulus that would correctly complete the sequence (see Figure 2). In this phase, each 5-element-long grammatical sequence was presented 5 times, balanced so that the missing element occurred once in each of the 5 positions within the sequence, for a total of 20 trials.

### Procedure

Experiment 1 took place in-person, in testing labs within the Institute of Neuroscience at Newcastle University. The experiment was coded using MATLAB and Psychtoolbox (Brainard and Vision, 1997; Kleiner et al., 2007; Pelli and Vision, 1997). Responses were made either with the mouse (in the Visual Recall and Sequence Completion tasks) or by pressing one of two keys on the keyboard (in the Grammaticality Judgement task).

Experiment 2 was an online replication of Experiment 1. The Visual Recall, Grammaticality Judgement and Sequence Completion tasks were adapted from MATLAB to PsychoPy (version 2021.2.3) to enable them to run online through Pavlovia. Participants completed the experiment on their own desktop or laptop computer.

As in traditional artificial grammar learning paradigms, the Visual Recall task was split into two phases: exposure and testing. In both phases, each sequence was presented serially across the screen (Figure 2). Each element was presented on the screen for 450 ms before being removed, and the elements were separated by an inter-element interval of 300 ms. In both exposure and testing phases, each sequence was separated by an inter sequence interval of 1,000 ms.

The experiment began with a baseline testing phase to assess working memory in each participant and familiarise participants with the task prior to any learning. This phase was identical to the other testing phases, except that it was not preceded by an exposure phase, and therefore we would predict no differences in recall accuracy between the ‘grammatical’ and ‘ungrammatical’ sequences.

#### Exposure phase

In the exposure phase, the participants were asked to pay attention to the sequences but were not asked to make any responses. Participant were not told about the presence of any patterns in the sequences. In the first exposure phase, following the baseline recall test, participants were presented with 64 grammatical sequences, consisting of 8 grammatical sequences repeated 8 times. This phase lasted approximately 5 min. Subsequent exposure phases were designed to refamiliarize the participants with the grammatical sequences, so were shorter, presenting each sequence 3 times (for a total of 24 sequences) and lasting approximately 2 min.

As Experiment 2 was completed online, attention checks were added to the exposure phase to ensure participants were paying attention to the sequences. One in eight of the exposure sequences was randomly selected to be an attention check sequence. In an attention check sequence, one element in the sequence was randomly selected and replaced with a star shape. Participants were instructed to attend to the sequences and to press the “space” key whenever they saw a star within a sequence. Seven participants were removed from the analysis for failing these attention checks.

#### Testing phase

In the testing phase, each testing sequence was presented in the same way as in the exposure phase. After the sequence was presented, there was a 1,000 ms retention period. Following this, the five stimulus elements were presented simultaneously, arranged in a circle on the screen (see Figure 2). The position of each element was randomised on each trial, so that participants could not rely on positional cues or motor sequence learning. The participant was asked to recreate the sequence by clicking on the appropriate elements in the correct order. No feedback was given. An inter-trial interval of 1,200 ms separated the participant’s response from the presentation of the next sequence. Participants completed 4 testing phases in total, each separated by a short exposure phase.

#### Grammaticality judgement task

After the Visual Recall task, the participants then completed the Grammaticality Judgement task. Prior to this task participants were re-familiarised with the grammatical sequences through a short exposure phase lasting 2 min, as described above. The participants were then informed that the sequences that they had just seen followed a pattern, and that they would be presented with sequences, some of which follow the same pattern and some that would not. The same 8 grammatical and 8 ungrammatical sequences that were used in the testing phase of the recall task were presented, in a random order. For this task, once the sequence was presented, participants were asked to judge if the sequence followed the pattern or not by pressing one of two keys (“C” or “M”) on a keyboard. Participants completed two runs of the Grammaticality judgement task, separated by a short 2-min refamiliarization phase.

#### Sequence completion task

In the Sequence Completion task, participants were presented with a 5-element-long grammatical sequence with one element missing and instructed to try to select the appropriate element to fill in the gap (see Figure 2). The Sequence Completion task consisted of 20 trials, and the sequences were counterbalanced so that for each of the four grammatical test sequences, the missing element occurred once in each of the 5 positions within the sequence.

### Data analysis

In the Visual Recall task, we calculated two measures of performance. The first method was to score a trial as correct if the participant recalled the entire sequence correctly (henceforth *absolute correct* score). The second method was to calculate the proportion of each sequence that was correctly recalled (henceforth *proportion correct* score). In the Grammaticality Judgement task, a trial was scored as correct if the participants successfully classified it as grammatical or ungrammatical, and performance on this task was compared to chance levels (50%). In the Sequence Completion task, a trial was scored as correct if the participant chose the correct element to complete the sequence. Note that, this task cannot be solved based on exclusion alone (i.e., assuming that if 4 shapes are present, then the correct answer must be the remaining shape) because the grammar allows for repetition of some stimuli. As only one element out of each 5-element long sequence was missing, chance performance was 20% in the Sequence Completion task.

In the online version of the task (Experiment 2), we assessed attention in the exposure phase by calculating the percentage of stars correctly identified in the probe exposure sequences (see Methods). As we expected that the majority of learning would occur in the longer initial exposure phase, it was particularly important to ensure that participants were paying attention in this block. Therefore, any participants who failed to respond to 7 out of 9 of the attention checks within the initial exposure phase were excluded from the analysis. In the subsequent shorter exposure phases, any participants who failed 2 out of 3 of the attention checks in more than one block were excluded from the analysis.

## Results

### Visual recall task

We predicted that recall accuracy would improve across testing blocks for the grammatical sequences relative to the ungrammatical sequences. As participants learned the statistical relationships between the elements we predicted higher levels of chunking, and thus recall, in the more predictable grammatical sequences. For both Experiments 1 and 2, we separately analysed the data based on both absolute correct and proportion correct scores using 2×4 repeated measures ANOVAs with factors: Condition (grammatical and ungrammatical) and Run (4 runs).

### Absolute correct analyses

**Figure 3:**
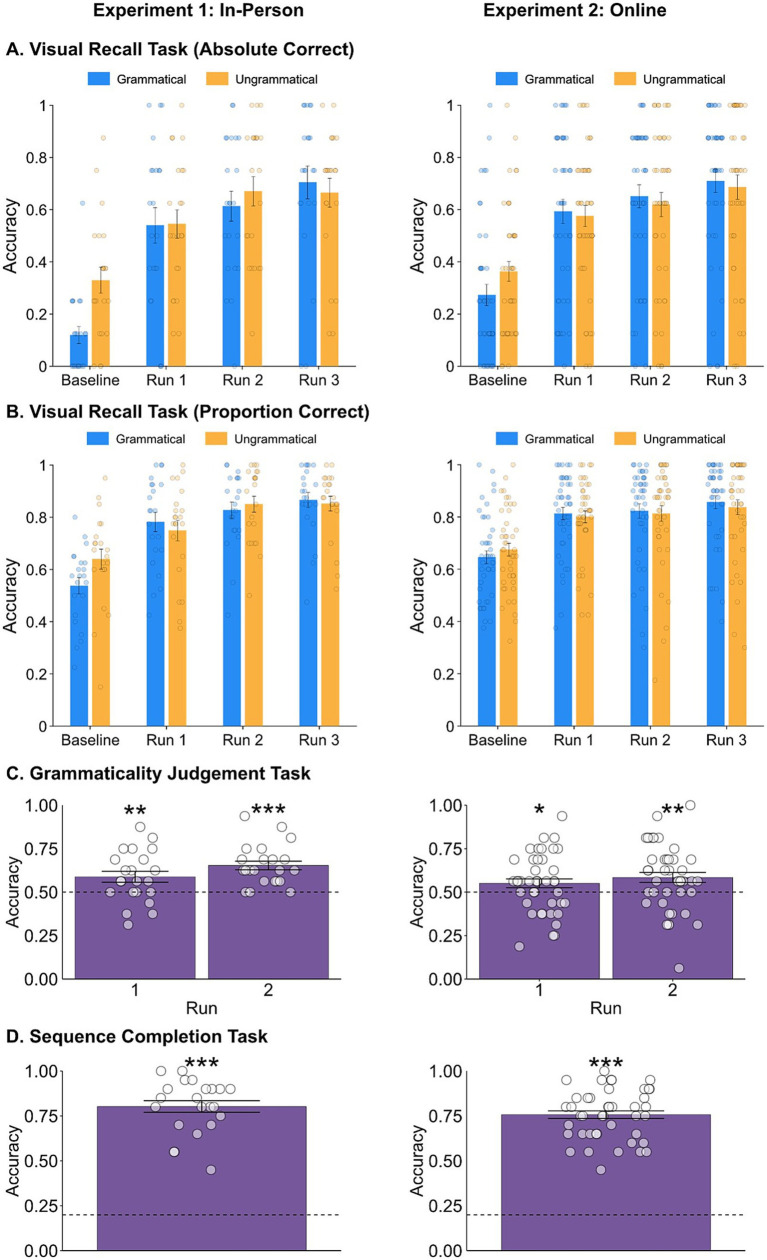
Results from Experiment 1 (in-person) are shown in the left panels and Experiment 2 (online) results are shown in the right panels. **(A,B)** Mean absolute and proportion recall accuracy for grammatical (blue) and ungrammatical (orange) sequences in the Visual Recall task. **(C)** Mean Grammaticality Judgement task accuracy across runs. Chance performance (50%) is indicated by a dashed line. **(D)** Mean Sequence Completion task accuracy. Chance performance (20%) is indicated by a dashed line. In all panels, individual performance is shown as white circles, and error bars represent ±1 SEM. Significance stars: *p < 0.05, **p < 0.01, ***p < 0.001.

Using absolute correct scores, in Experiment 1 (Figure 3A), there was a main effect of Run (*F*_3, 63_ = 47.389, *p* < 0.001), indicating an improvement in recall accuracy of both grammatical and ungrammatical sequences over the course of the experiment. Post-hoc tests (Bonferroni corrected) indicated significant differences in recall accuracy between the baseline run and subsequent testing runs (*p* < 0.001 in all cases), and between testing run 1 and the final testing run (*p* = 0.006), but not between testing run 1 and testing run 2 (*p* = 0.101). There were no significant differences in recall accuracy between other runs (*p* > 0.05). Moreover, there was a small but significant main effect of Condition, however this indicated that recall of grammatical strings was poorer than ungrammatical strings (*F*_1, 21_ = 6.73, *p* = 0.017). We also found a significant interaction between Condition and Run (*F*_3, 63_ = 6.09, *p* = 0.001), indicating that recall of grammatical sequence improved across runs to a greater extent than recall of ungrammatical sequences.

The absolute correct scores in Experiment 2 replicated these results (Figure 3A). There was a main effect of Run (*F*_3, 87.58_ = 41.38, *p* < 0.001). Bonferroni corrected post-hoc tests indicated significant differences in recall accuracy between the baseline run and testing runs 1, 2 and 3 (*p* < 0.001), between testing run 1 and testing run 3 (*p* = 0.013) but not between testing run 1 and testing run 2 (*p* = 0.454). There were no significant differences in recall accuracy between other runs (*p* > 0.05). There was no main effect of Condition (*F*_1, 42_ = 0.056, *p* = 0.814) meaning that grammatical sequences were not recalled better than ungrammatical sequences. However, again there was a significant interaction between Condition and Run (*F*_3, 126_ = 3.77, *p* = 0.012), with grammatical performance again increasing more than ungrammatical performance.

### Proportion correct analyses

In both experiments, similar patterns of results were observed when using proportion correct scores (Figure 2B). In Experiment 1 there was a main effect of Run (*F*_2.23, 46.74_ = 41.10, *p* < 0.001). Post-hoc tests (Bonferroni corrected) indicated similar significant differences in recall accuracy between the baseline run and subsequent testing runs (*p* < 0.001), and between testing run 1 and the final testing run (*p* = 0.004), but not between testing run 1 and testing run 2 (*p* = 0.053). There were no significant differences in recall accuracy between other runs (*p* > 0.05). There was no main effect of Condition (*F*_1,21_ = 2.07, *p* = 0.165), but again there was an interaction between Run and Condition (*F*_2.17, 45.47_ = 6.79, *p* = 0.002). In Experiment 2, there was a main effect of Run (*F*_3,80_ = 34.76, *p* < 0.001). Post-hoc tests (Bonferroni corrected) showed significant differences in recall accuracy between the baseline run and subsequent testing runs (*p* < 0.001), but no significant differences between other testing runs (*p* > 0.05). There was no main effect of Condition (*F*_1, 42_ = 0.088, *p* = 0.768), and in this case no interaction between Run and Condition (*F*_3, 126_ = 1.88, *p* = 0.136).

Across both experiments, using both absolute and proportion correct scores, we did not find the predicted learning effect (main effect of Condition) in the Visual Recall task. There was some evidence of an interaction between Condition and Run across experiments (in 3 out of 4 of the analyses). However, this cannot be interpreted as evidence of learning as we predicted because this effect was driven by particularly *poor* recall accuracy of grammatical sequences in the baseline block (seen in Figure 3). As the baseline block occurred before any exposure to grammatical sequences had taken place, it is the one block we would not predict any differences in recall between grammatical and ungrammatical sequences (see Discussion for possible explanations of this effect).

### Grammaticality judgement task

In Experiment 1, participants correctly classified the testing sequences as grammatical or ungrammatical across both runs of the Grammaticality Judgement task (run 1: *M* = 0.59, SEM = 0.031; *t*_21_ = 2.796, *p* = 0.005; run 2: *M* = 0.65, SEM = 0.025; *t*_21_ = 6.156, *p* < 0.001; Figure 3C). There was some indication that performance improved across runs, however this difference did not reach significance (*t*_21_ = 1.879, *p* = 0.074).

In Experiment 2, participants performed above chance in run 1 (*M* = 0.55, SEM = 0.03; *t_42_* = 2.01, *p* = 0.026) and run 2 (*M* = 0.58, SEM = 0.03; *t*_42_ = 2.93, *p* = 0.003; Figure 3C). A paired *t* test showed no difference in performance between runs (*t*_42_ = 1.15, *p* = 0.257). These findings suggest that the grammaticality judgement task reveals some evidence of learning that was not observed during the visual recall task.

### Sequence completion task

In the Sequence Completion task, participants’ performance was compared to chance (20%) using a one sample *t*-test. In Experiment 1, participants were significantly more likely to choose the correct element to fill in the gap (*t*_21_ = 18.835, *p* < 0.001; Figure 3D). This was also reflected in Experiment 2, where performance on the Sequence Completion task was also above chance (*t*_42_ = 26.33, *p* < 0.001; Figure 3D).

### Implicit and explicit processing

Although we planned to correlate performance across the Visual Recall task (using difference scores for recall of grammatical and ungrammatical sequences) with performance in the subsequent explicit tasks, the lack of a learning effect in the Visual Recall task means that such analyses are unlikely to be informative. For completeness, the correlations are reported in Tables 1, 2 for the in-person and online experiments, respectively. No correlations were found between Visual Recall task performance and performance in the reflection-based tasks.

**Table 1:** Correlation matrix for Experiment 1.

	Testing run 1 (G-UG)	Testing run 2 (G-UG)	Testing run 3 (G-UG)	AFC run 1	AFC run 2
Testing run 2 (G-UG)	*r* = 0.142 *p* = 0.527				
Testing run 3 (G-UG)	*r* = 0.146 *p* = 0.515	*r* = 0.33 *p* = 0.134			
AFC run 1	*r* = 0.227 *p* = 0.309	*r* = −0.391 *p* = 0.072	*r* = −0.17 *p* = 0.449		
AFC run 2	*r* = 0.377 *p* = 0.083	*r* = −0.185 *p* = 0.41	*r* = 0.064 *p* = 0.778	*r* = 0.258 *p* = 0.247	
Sequence completion	*r* = 0.328 *p* = 0.136	*r* = 0.148 *p* = 0.51	*r* = 0.34 *p* = 0.121	*r* = 0.145 *p* = 0.52	***r* = 0.531** ***p* = 0.011**

Bold values indicate significant correlations.

**Table 2:** Correlation matrix for Experiment 2.

	Testing run 1 (G-UG)	Testing run 2 (G-UG)	Testing run 3 (G-UG)	AFC run 1	AFC run 2
Testing run 2 (G-UG)	*r* = 0.215 *p* = 0.166				
Testing run 3 (G-UG)	*r* = 0.155 *p* = 0.32	*r* = 0.194 *p* = 0.214			
AFC run 1	*r* = 0.187 *p* = 0.23	*r* = 0.142 *p* = 0.363	*r* = −0.108 *p* = 0.49		
AFC run 2	*r* = 0.11 *p* = 0.481	*r* = −0.046 *p* = 0.772	*r* = −0.039 *p* = 0.803	***r* = 0.427** ***p* = 0.004**	
Sequence completion	*r* = 0.097 *p* = 0.535	*r* = 0.108 *p* = 0.491	*r* = 0.081 *p* = 0.606	*r* = 0.214 *p* = 0.169	***r* = 0.635** ***p* < 0.001**

Bold values indicate significant correlations.

While there were no correlations between Visual Recall task performance and any of the other measures (see Tables 1, 2), the Sequence Competition task did correlate with the second run of the Grammaticality Judgement task in both experiments. This suggests that performance on the two reflection-based tasks appeared to be related to one another, but not to the processing-based recall measure.

### In-person vs. online performance

To compare performance between Experiment 1 (in-person) and Experiment 2 (online), we added the between-subjects factor of Task (in-person or online) to the previous ANOVAs using both absolute and proportion correct scores. In the Visual Recall task, we found no main effect of Task when using absolute correct scores (*F*_1, 63_ = 0.391, *p* = 0.534), and no interactions between Task and Run (*F*_3, 189_ = 1.072, *p* = 0.362), Task and Grammaticality (*F*_1, 63_ = 3.155, *p* = 0.081), or Task, Run and Grammaticality (*F*_3, 189_ = 1.417, *p* = 0.239). When using proportion correct scores, we found no main effect of Task (*F*_1, 63_ = 0.336, *p* = 0.564), and no interaction between Task and Grammaticality (*F*_1, 63_ = 1.701, *p* = 0.197), or Task, Run and Grammaticality (*F*_3, 189_ = 2.085, *p* = 0.104). However, there was a significant interaction between Task and Run (*F*_3, 189_ = 3.360, *p* = 0.02), which reflected a greater improvement in recall for all sequences across runs in the in-person task over the online task.

We also compared in-person and online performance in the Grammaticality Judgement task using a 2×2 mixed ANOVA, with Run (2 runs) as a within-subjects factor and Task (in-person or online) as a between-subjects factor. We found no main effect of Task (*F*_1, 63_ = 2.20, *p* = 0.143), and no interaction between Task and Run (*F*_1, 63_ = 0.448, *p* = 0.506). An independent samples *t-*test showed no difference between in-person and online performance in the Sequence Completion task (*t*_63_ = 1.21, *p* = 0.230).

## Discussion

In two experiments, contrary to our predictions we found no evidence of implicit statistical learning in the Visual Recall task. We did not observe the predicted increase in recall accuracy for the grammatical sequences over the ungrammatical sequences. Instead, we saw a suggestion of the opposite effect in the baseline period. It appears that this interaction effect was driven by participants’ responses to sequences that contained repetition of any sequence element within a given sequence. As all of the testing sequences were five elements long, and the experiment used five different stimuli, despite presenting participants with sequences containing repetitions during the baseline recall testing, they were very reluctant to select stimuli to create sequences that contained repetition (e.g., A *C* F *C* G), instead preferring to use each element exactly once per sequence. Due to the design of the stimuli (see Wilson et al., 2015; Milne et al., 2018), the grammatical sequences contained more stimulus repetitions than did the ungrammatical sequences, which led to more participants making these types of recall errors on the grammatical trials. In the subsequent exposure phases, participants observed many more sequences containing stimulus repetitions. This bias toward avoiding repetitions disappeared, removing this difference in recall accuracy between grammatical and ungrammatical sequences, and leading to a relative increase in grammatical performance. Thus, we cannot conclude that the significant effects reported here are due to learning of the artificial grammar. This lack of learning measured during the Visual Recall task occurred despite observing small but significant learning effects in the subsequent reflection-based measures: we found evidence of learning in both the Grammaticality Judgement and Sequence Completion tasks. The results across all tasks were highly consistent between Experiments 1 and 2.

There are two possible explanations for why there was evidence of learning in the explicit tasks, but not the Visual Recall task. Firstly, it is possible that learning did occur during the Visual Recall task, but this was not reflected in improved recall performance. Alternatively, it is possible that learning did not occur until *after* the Visual Recall task, when participants were told about the presence of rules and had the opportunity to complete an exposure phase with this knowledge. While the current experiments are not able to disentangle these possible explanations, previous studies using identical exposure phases have elicited immediate learning, as evidenced by a grammaticality judgement task (Milne et al., 2018). Therefore, it is possible that learning occurred from the outset, but that our Visual Recall task was unable to measure evidence of this learning. We also saw significant learning from the first run of the Grammaticality Judgement task in both experiments, although, again, it is impossible to demonstrate whether this learning occurred in the immediately preceding exposure phase, or during the Visual Recall task.

There are several methodological reasons that may explain why serial recall was not an effective measure of learning in this experiment. The first relates to the predictability of the ungrammatical sequences. The rationale for the use of serial recall as a measure of statistical learning is that when items consistently co-occur in sequences, it becomes cognitively efficient to chunk them into a single unit (as with phonemes into words, Saffran et al., 1996). Most previous studies involved the learning of within word transitions which are 100% predictable: that is, the transitional probabilities within a “chunk” are 1.0 (Saffran et al., 1996; Isbilen et al., 2017). The goal of this experiment was to assess whether a similar effect would be observed for less predictable statistical relationships, such as those present in the artificial grammar used here. Certain transitions between elements are more common than others in the exposure sequences, so we hypothesised that these transitions might be more easily chunked, and thus recalled. However, we did not find any evidence of learning in the Visual Recall task in either experiment. This may suggest that, contrary to our predictions, that visual recall tasks are only effective measures of statistical learning when the transitions between elements within a chunk are 100% predictable. Despite this, the learning observed in the subsequent reflection-based tasks suggests that learning of the relationships between elements did occur. Therefore, the lack of learning in the Visual Recall task may have been due to issues with the design of the task itself. Based on these data, it is not possible to conclude whether serial visual recall is an effective measure of statistical learning of more variable transitions, and future research should provide more clarity on this issue.

A second possible explanation for the lack of learning observed in the Visual Recall task may be related to the predictability of the ungrammatical sequences in these experiments. We predicted that we would see improved recall of grammatical sequences, because they are more predictable and therefore have higher transitional probabilities than the ungrammatical sequences. However, in this experiment, the ungrammatical sequences contained subtle violations designed to identify the specific features of the grammar that participants may have learned (Wilson et al., 2015; Milne et al., 2018). Therefore, the mean transitional probabilities of the ungrammatical sequences (Figure 1) were higher than those used in previous auditory serial recall tasks, which consisted of random transitions (Isbilen et al., 2017, 2020, 2022; Kidd et al., 2020). Therefore, our ungrammatical sequences consist primarily of legal transitions that can be chunked, facilitating recall of the majority of the ungrammatical sequence in the same way as the grammatical sequences, with relatively few unexpected (ungrammatical) transitions. This may also explain why we do not see any differences in recall of grammatical and ungrammatical sequences in this experiment. It is therefore possible that recall may only be an effective measure of learning when the ungrammatical sequences consist primarily of illegal transitions.

Finally, to allow us to measure learning throughout the task, ungrammatical sequences were interspersed within each Recall Block. However, this may have interfered with learning of the grammar. In addition to the grammatical transitions shown in the exposure and recall phases of the Visual Recall task, participants also saw ungrammatical transitions in the Recall phase. This lowers the average transitional probabilities that they are exposed to compared with traditional paradigms. This is particularly relevant as participants may have been more attentive due to the presence of an active task during the Recall Blocks compared to the exposure phases, resulting in ungrammatical transitions being more salient than in previous experiments.

The aim of these experiments was to develop a processing-based measure of implicit statistical learning based on previous visual artificial grammar learning paradigms, and to combine it with more traditional reflection-based tasks to investigate the nature of the knowledge acquired during implicit statistical learning. Specifically, we assessed whether sequences containing more variable transitions between stimulus elements would elicit increases in recall accuracy similar to sequences containing much more predictable, fixed transitions (as in Isbilen et al., 2017, 2020; Kidd et al., 2020). Although we found no evidence of learning in the Visual Recall tasks, we did replicate prior learning effects using subsequent reflection-based measures. Based on this reflection-based learning, and the successful use of serial recall as a measure of implicit statistical learning in previous studies (Isbilen et al., 2017, 2020; Kidd et al., 2020), the lack of learning in the Visual Recall tasks here is likely due to methodological factors. These might include the design of the Visual Recall task, the predictability of the transitions within the grammar being learned, and the predictability of the ungrammatical sequences. If this is the case, suggests that while this specific serial recall task did not measure learning, this does not reflect an inability to measure learning using serial recall more generally. These experiments highlight a number of methodological factors that may influence the extent to which implicit statistical learning can be measured using serial recall. Future research should focus on the systematic manipulation of these factors in order to determine how serial recall can be utilised to best reflect implicit statistical learning. In particular, it is important to assess the extent to which serial recall can be used to measure implicit statistical learning of more variable relationships, as opposed to highly predictable dependencies.

## Data Availability

The datasets presented in this study can be found in online repositories. The names of the repository/repositories and accession number(s) can be found at: https://osf.io/dpeu8/.
